# Comparative Prognostic Performance of 18 Routine Laboratory-Derived Indices for In-Hospital Mortality in Patients with COVID-19

**DOI:** 10.3390/jcm15218419

**Published:** 2026-10-29

**Authors:** Mehmet Serdar Yıldırım, Ferhat Bingöl, Süleyman Özçaylak, Nizam Demir, Selman Çetin, Sedat Çiçek, Jehat Kılıç

**Affiliations:** 1 Department of Internal Medicine, Gazi Yaşargil Training and Research Hospital, Diyarbakır 21000, Türkiye; yildirs21@gmail.com (M.S.Y.); fbingol2184@gmail.com (F.B.); ozcaylaksuleyman@gmail.com (S.Ö.); nzmdmr21@gmail.com (N.D.); 2 Department of Gastroenterology, Fırat University Faculty of Medicine, Elazig 23119, Türkiye; drselmancetn@gmail.com (S.Ç.); drsedatcicek23@gmail.com (S.Ç.); 3 Department of Rheumatology, Fırat University Faculty of Medicine, Elazig 23119, Türkiye

**Keywords:** COVID-19, in-hospital mortality, inflammatory indices, C-reactive protein-to-albumin ratio, prognostic nutritional index, risk stratification

## Abstract

**Background/Objectives:** Numerous hematologic, inflammatory, biochemical, and nutritional indices have been proposed as prognostic markers in coronavirus disease 2019 (COVID-19), but direct comparison of a broad range of these indices within the same cohort remains limited. This study aimed to evaluate and compare the prognostic performance of multiple routinely available indices for in-hospital mortality. **Methods:** This retrospective observational cohort included 1952 adults hospitalized with COVID-19. Demographic, clinical, comorbidity, and laboratory data were obtained from medical records. Eighteen derived indices were evaluated, including NLR, PLR, SII, CAR, CLR, LAR, UAR, CPR, DAR, and PNI. Receiver operating characteristic analysis was performed to assess discrimination for in-hospital mortality, and optimal cut-offs were determined using the Youden index. Selected indices were further evaluated in separate multivariable logistic regression models adjusted for age, sex, diabetes mellitus, CAD/IHD, and eGFR. **Results:** In-hospital mortality occurred in 80 patients (4.1%). Non-survivors were older and had higher inflammatory and biochemical markers and lower lymphocyte counts and albumin levels. CAR and CLR showed the highest numerical AUCs for in-hospital mortality (0.870 for both), followed by PNI (0.857), CPR (0.852), DAR (0.829), UAR (0.823), LAR (0.815), and NLR (0.807). In separate multivariable models adjusted for age, sex, diabetes mellitus, CAD/IHD, and eGFR, ln(CLR), ln(CAR), ln(CPR), ln(LAR), ln(UAR), ln(NLR), ln(dNLR), and ln(DAR) remained independently associated with mortality (all *p* < 0.001). Among the adjusted models, the CLR model showed the highest explained variance (Nagelkerke R^2^ = 0.511) with good calibration (Hosmer–Lemeshow *p* = 0.997); ln(CLR) remained independently associated with mortality (aOR 3.590, 95% CI 2.517–5.120; *p* < 0.001). **Conclusions:** Several routinely available composite indices were associated with in-hospital mortality in COVID-19. Among the evaluated indices, CAR and CLR showed the highest individual discrimination, while the CLR-based adjusted model showed the most favorable model-fit profile among the multivariable models evaluated. Multiple selected indices remained independently associated with mortality after adjustment for clinical covariates. These findings suggest that such markers may have potential value as adjunctive tools for mortality risk stratification.

## 1. Introduction

Coronavirus disease 2019 (COVID-19) has a highly heterogeneous clinical course, ranging from mild or moderate disease to severe pneumonia, respiratory failure, multiorgan dysfunction, and death. Although the acute phase of the pandemic has passed, COVID-19 provided a unique clinical setting in which systemic inflammation, immune dysregulation, endothelial injury, coagulation abnormalities, and nutritional deterioration could be evaluated simultaneously. Early identification of patients at increased risk of mortality therefore became an important component of clinical risk stratification [1,2].

Routine hematologic and biochemical parameters have been widely investigated for this purpose because they are inexpensive, rapidly available, and can be obtained at the time of hospital admission. Among the most frequently studied markers, the neutrophil-to-lymphocyte ratio (NLR) has emerged as a simple indicator of the balance between innate inflammatory activation and adaptive immune suppression. Meta-analyses have consistently demonstrated that higher admission NLR values are associated with both greater COVID-19 severity and increased mortality [1,2]. Similarly, the systemic immune-inflammation index (SII), which integrates neutrophil, platelet, and lymphocyte counts, has been associated with severe disease and mortality and may provide broader information regarding systemic inflammatory activity [3].

In addition to cell-count-derived indices, composite markers incorporating inflammatory, biochemical, and nutritional variables have gained increasing attention. C-reactive protein-to-albumin ratio (CAR) combines an acute-phase reactant with a negative acute-phase and nutritional marker and may therefore reflect both inflammatory burden and physiological reserve. Ratios incorporating urea, lactate dehydrogenase, D-dimer, fibrinogen, and albumin may likewise capture different aspects of renal dysfunction, tissue injury, coagulation activation, inflammation, and nutritional impairment. For example, an elevated blood urea nitrogen-to-albumin ratio has been associated with increased in-hospital mortality in patients with COVID-19 [4].

Nutritional and immunological status may also be closely related to COVID-19 outcomes. The prognostic nutritional index (PNI), derived from serum albumin and lymphocyte count, has been evaluated as a simple measure of immunonutritional status. Lower PNI values have been associated with increased mortality among patients with COVID-19, and pooled evidence has supported its potential value for prognostic assessment [5,6].

Despite the large number of proposed biomarkers, most previous studies have focused on a single index or a relatively limited group of parameters. Direct head-to-head comparison of a broad spectrum of hematologic, inflammatory, biochemical, and nutritional indices within the same COVID-19 population remains less common. Such an approach may be useful for identifying which readily available indices provide the greatest discriminatory ability for mortality and whether their associations persist after adjustment for relevant clinical factors.

Therefore, the present study aimed to comprehensively evaluate 18 routinely calculable hematologic, inflammatory, biochemical, and nutritional indices in hospitalized patients with COVID-19. We compared their ability to discriminate in-hospital mortality using receiver operating characteristic analysis and further assessed whether selected indices remained independently associated with mortality after adjustment for demographic characteristics and relevant comorbidities.

## 2. Materials and Methods

### 2.1. Study Design and Population

This retrospective observational cohort study included adult patients admitted to Gazi Yaşargil Training and Research Hospital between 16 March 2020 and 4 July 2020. COVID-19 was confirmed by detection of SARS-CoV-2 RNA using reverse transcription polymerase chain reaction (RT-PCR). Adult patients aged ≥18 years who were hospitalized with laboratory-confirmed SARS-CoV-2 infection and whose hospitalization was primarily related to COVID-19 were eligible for inclusion. Patients with insufficient admission laboratory data required for calculation of the investigated indices were excluded. When more than one eligible hospitalization occurred for the same patient during the study period, only the first hospitalization was included in the analysis.

A total of 1952 patients were included in the final study population. The primary outcome was in-hospital mortality, defined as death occurring during the index hospitalization. Patients were therefore classified as survivors or non-survivors according to their status at hospital discharge.

Demographic characteristics, duration of hospitalization, intensive care unit admission, and comorbidities were obtained from the hospital records. Recorded comorbidities included hypertension, diabetes mellitus, coronary artery disease/ischemic heart disease (CAD/IHD), asthma, chronic obstructive pulmonary disease (COPD), and other comorbid conditions. Comorbidity information was available for 1199 patients.

### 2.2. Laboratory Measurements

Laboratory values obtained at hospital admission were extracted from the electronic medical records and used for the analyses. These included white blood cell count, neutrophil count, lymphocyte count, hemoglobin, hematocrit, platelet count, red cell distribution width coefficient of variation (RDW-CV), mean platelet volume (MPV), glucose, urea, creatinine, sodium, potassium, calcium, albumin, aspartate aminotransferase (AST), alanine aminotransferase (ALT), lactate dehydrogenase (LDH), and C-reactive protein (CRP), together with the laboratory variables required for calculation of the investigated composite indices.

### 2.3. Hematologic and Inflammatory Indices

The following hematologic, inflammatory, and nutritional indices were evaluated: neutrophil-to-lymphocyte ratio (NLR), platelet-to-lymphocyte ratio (PLR), systemic immune-inflammation index (SII) [7], neutrophil-to-platelet ratio (NPR), RDW-to-platelet ratio (RPR), MPV-to-platelet ratio (MPR), derived neutrophil-to-lymphocyte ratio (dNLR), CRP-to-albumin ratio (CAR), CRP-to-lymphocyte ratio (CLR), LDH-to-albumin ratio (LAR), urea-to-albumin ratio (UAR), LDH-to-lymphocyte ratio (LLR), CRP-to-platelet ratio (CPR), RDW-to-lymphocyte ratio (RLR), fibrinogen-to-albumin ratio (FAR), D-dimer-to-albumin ratio (DAR), prognostic nutritional index (PNI), and AST/ALT ratio.

PNI was calculated as serum albumin (g/L) + 5 × peripheral blood lymphocyte count (×10^9^/L). The derived neutrophil-to-lymphocyte ratio was calculated as dNLR = neutrophil count/(white blood cell count—neutrophil count), with both cell counts expressed as ×10^9^/L. The AST/ALT ratio was calculated by dividing AST by ALT. PNI was calculated using serum albumin and peripheral blood lymphocyte count [8], and the AST/ALT ratio was calculated by dividing AST by ALT.

### 2.4. Statistical Analysis

Statistical analyses were performed using IBM SPSS Statistics version 26.0 (IBM Corp., Armonk, NY, USA). Distributional characteristics of continuous variables were evaluated using appropriate normality tests together with visual assessment of their distributions. Because most laboratory variables and inflammatory indices showed non-normal distributions, continuous data were summarized as median (interquartile range [IQR]). Categorical variables were expressed as numbers and percentages. Continuous variables were compared between survivors and non-survivors using the Mann–Whitney U test. Categorical variables were compared using Pearson’s chi-square test, while Fisher’s exact test was used when expected cell frequencies were insufficient. The association between clinical variables and in-hospital mortality was initially investigated using univariate binary logistic regression analysis. Crude odds ratios (ORs) with 95% confidence intervals (CIs) were reported. Because several inflammatory indices displayed marked right-skewness, natural logarithmic transformation was applied before logistic regression modeling. Both original-scale and natural-log-transformed indices were examined in univariate analyses. Patients with non-positive values that could not undergo logarithmic transformation were excluded only from the corresponding transformed analysis.

During the preparation of this manuscript, the authors used ChatGPT (OpenAI) to assist with language editing and the identification of the potentially relevant literature. All references and AI-assisted content were independently reviewed and verified by the authors, who take full re-sponsibility for the accuracy and integrity of the final manuscript.

#### 2.4.1. ROC Analysis

The discriminatory ability of the inflammatory indices for in-hospital mortality was evaluated using receiver operating characteristic (ROC) curve analysis. The area under the ROC curve (AUC) was used to quantify discrimination. Optimal cut-off values were determined according to the maximum Youden index [9], calculated as:

Youden index = sensitivity + specificity − 1

Sensitivity and specificity corresponding to each optimal cut-off were reported.

#### 2.4.2. Multivariable Logistic Regression

To assess whether selected laboratory-derived indices were independently associated with in-hospital mortality, separate multivariable binary logistic regression models were constructed. Because several of the investigated indices share common laboratory components and were therefore expected to exhibit substantial collinearity, they were not entered simultaneously into the same regression model. Instead, each selected index was evaluated in a separate model adjusted for age, sex, diabetes mellitus, coronary artery disease/ischemic heart disease (CAD/IHD), and estimated glomerular filtration rate (eGFR). The indices evaluated in the adjusted models were CLR, CAR, CPR, LAR, UAR, NLR, dNLR, and DAR. eGFR was calculated using the 2021 CKD-EPI creatinine equation and was entered as a continuous variable.

Missing data were handled using a model-specific complete-case approach. Thus, each multivariable analysis included only patients with available data for age, sex, diabetes mellitus, CAD/IHD, eGFR, and the laboratory-derived index included in that particular model. Because comorbidity information and some laboratory measurements were not available for all patients, the analytical sample size varied across models, ranging from 1115 to 1198 patients, with 50–61 in-hospital deaths. Model-specific sample sizes and numbers of mortality events are reported in Table 4. Missing comorbidity information was treated as missing and was not interpreted as absence of the corresponding condition. Right-skewed laboratory-derived indices were natural logarithm-transformed before inclusion in the regression models. Results were expressed as adjusted odds ratios (aORs) with 95% confidence intervals (CIs). Model performance and calibration were evaluated using the −2 log likelihood, Nagelkerke R^2^, and Hosmer–Lemeshow goodness-of-fit test. All statistical tests were two-sided, and a *p* value <0.05 was considered statistically significant.

### 2.5. Ethical Considerations

The study protocol was approved by the Ethics Committee of Gazi Yaşargil Training and Research Hospital (approval no. 405, dated 14 July 2026). The study was conducted in accordance with the principles of the Declaration of Helsinki. Given the retrospective nature of the study, the requirement for written informed consent was waived by the ethics committee.

## 3. Results

### 3.1. Baseline Characteristics

A total of 1952 hospitalized patients with COVID-19 were included. The median age was 48 years (IQR, 35–61), and 978 patients (50.1%) were male. ICU admission occurred in 176 patients (9.0%), and 80 patients (4.1%) died during hospitalization. Comorbidity information was available for 1199 patients; hypertension was the most frequent comorbidity (25.2%), followed by CAD/IHD (11.3%) and diabetes mellitus (9.8%). Median CRP was 18.0 mg/L (IQR, 3.8–53.8), albumin 39 g/L (IQR, 36–42), and LDH 242 U/L (IQR, 197–305). Among the derived indices, median NLR was 2.82 (IQR, 1.88–4.40), CAR 0.45 (IQR, 0.10–1.46), CLR 13.10 (IQR, 2.81–44.41), and PNI 46.5 (IQR, 42.4–51.1) (Table 1).

### 3.2. Comparison Between Survivors and Non-Survivors

Non-survivors were substantially older than survivors (72.5 vs. 47 years, *p* < 0.001) and more frequently had hypertension, diabetes mellitus, and CAD/IHD. They also had higher WBC and neutrophil counts, glucose, urea, creatinine, RDW-CV, AST, LDH, and CRP levels, together with lower lymphocyte counts, hemoglobin, hematocrit, sodium, calcium, and albumin levels. Sex, platelet count, MPV, potassium, ALT, asthma, and COPD did not differ significantly between groups (Table 2). Univariate associations of demographic and comorbidity variables with mortality are provided in Supplementary Table S2, while univariate analyses of the derived indices on the log-transformed and original scales are presented in Supplementary Tables S3 and S4, respectively. Model-fit characteristics of the univariate log-transformed models are shown in Supplementary Table S5.

### 3.3. Discrimination of Laboratory-Derived Indices

**Figure 1:**
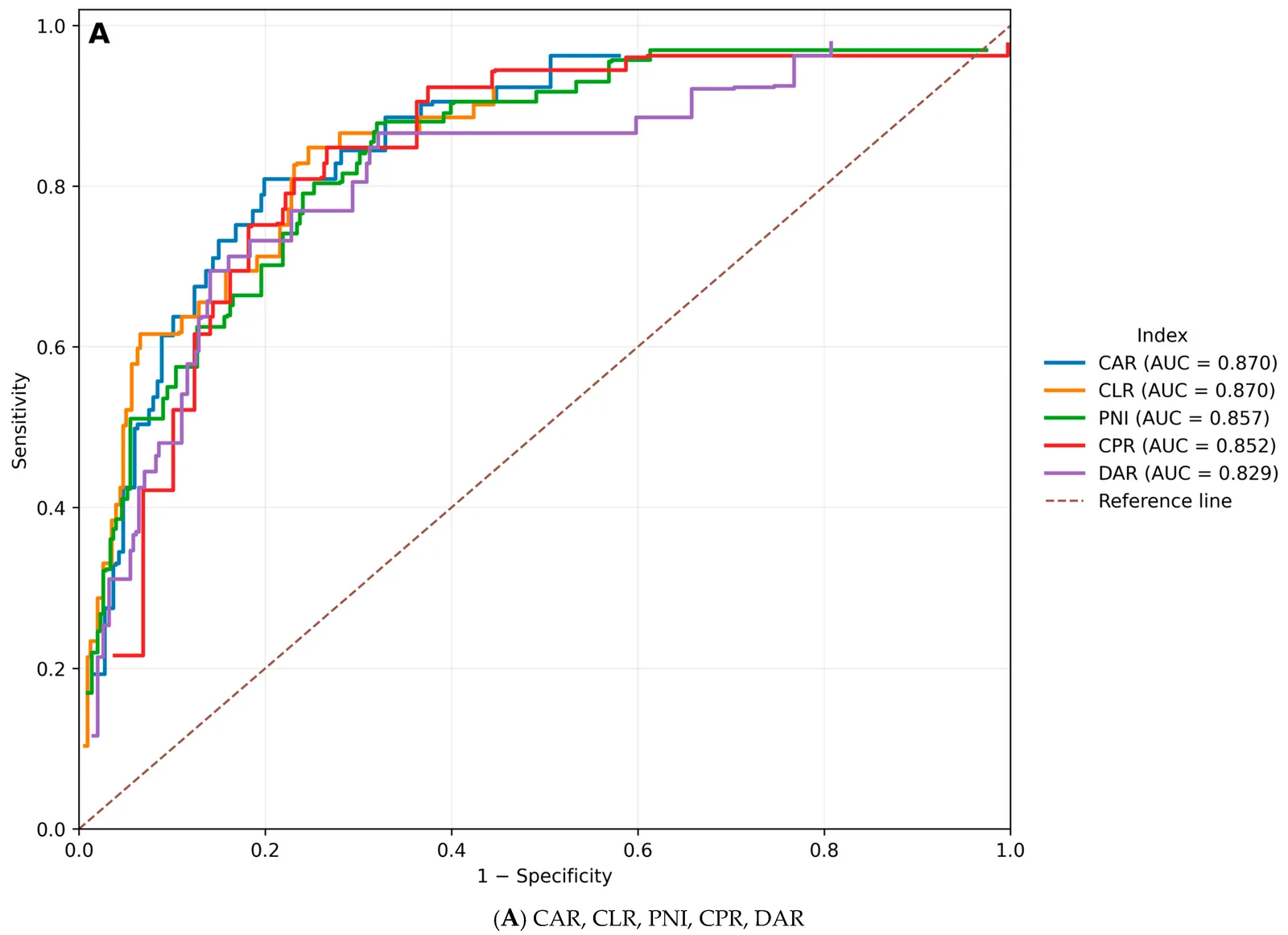

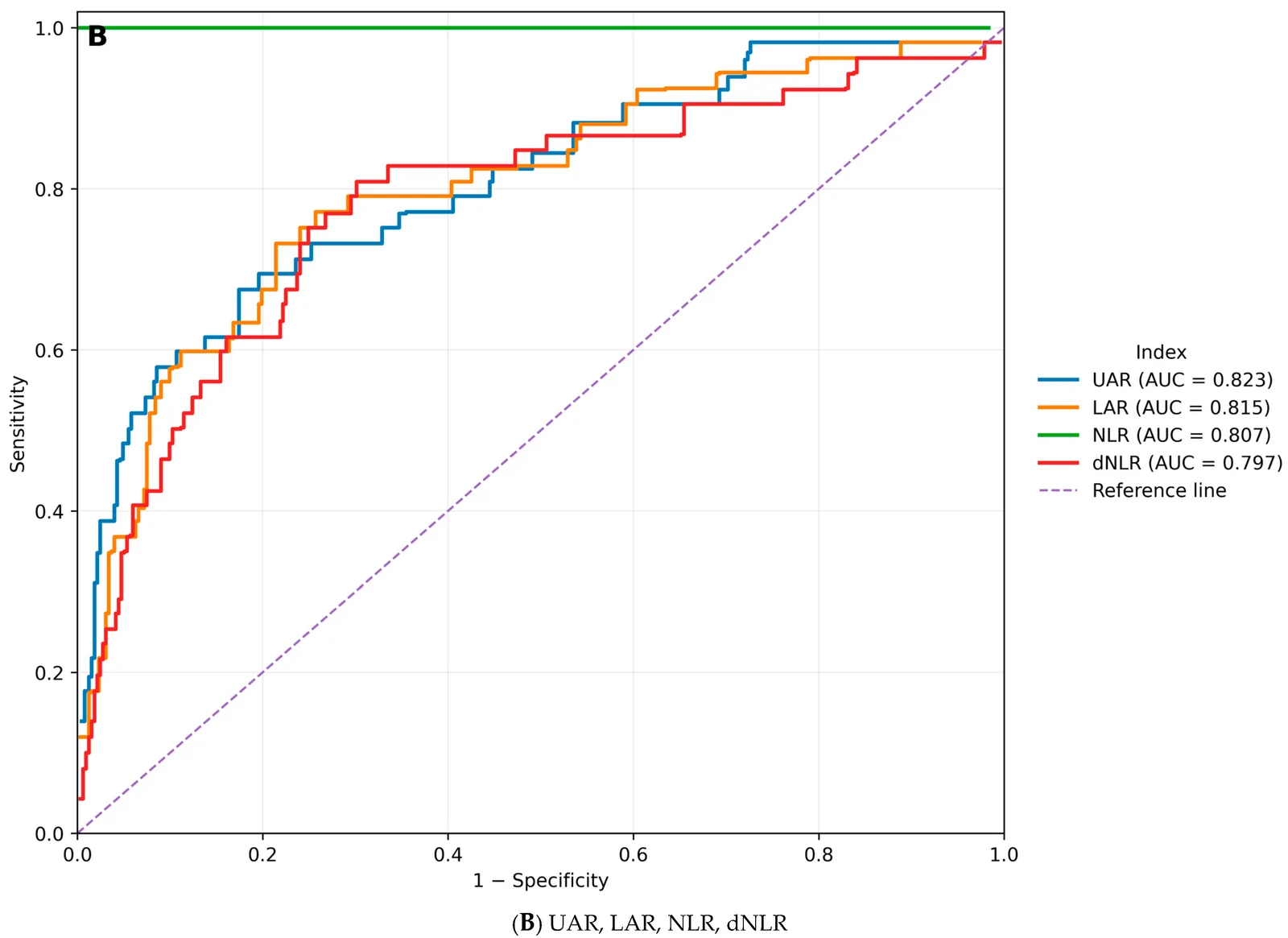
Receiver operating characteristic (ROC) curves of selected laboratory-derived indices for predicting in-hospital mortality in hospitalized patients with COVID-19. (**A**) Higher-performing indices: CAR, CLR, PNI, CPR, and DAR. (**B**) Additional indices: UAR, LAR, NLR, and dNLR.

In the available-case ROC analyses, CAR and CLR had the highest numerical AUCs for in-hospital mortality (both 0.870), followed by PNI (0.857), CPR (0.852), DAR (0.829), UAR (0.823), LAR (0.815), NLR (0.807), and dNLR (0.797). RPR and MPR showed little discriminatory ability, with AUCs of 0.519 and 0.501, respectively. The corresponding Youden-derived thresholds, sensitivities, and specificities are presented in Table 3, and selected ROC curves are displayed in Figure 1.

Because the indices had slightly different numbers of available observations, formal comparisons of the leading indices were additionally performed in a common complete-case cohort of 1934 patients, including 76 deaths and 1858 survivors. In this cohort, the AUC was 0.888 for CLR, 0.886 for CAR, 0.862 for CPR, and 0.856 for PNI. Pairwise DeLong testing showed no significant difference between CLR and CAR (*p* = 0.864), CLR and PNI (*p* = 0.088), CAR and PNI (*p* = 0.118), or CPR and PNI (*p* = 0.777). CLR and CAR showed significantly greater discrimination than CPR (*p* = 0.014 and *p* = 0.005, respectively) (Supplementary Table S9).

### 3.4. Multivariable Analyses

In separate multivariable models adjusted for age, sex, diabetes mellitus, CAD/IHD, and eGFR, all eight selected log-transformed indices remained independently associated with in-hospital mortality (all *p* < 0.001). The adjusted ORs were 17.216 for ln(LAR), 4.399 for ln(CAR), 4.195 for ln(UAR), 3.590 for ln(CLR), 3.552 for ln(CPR), 3.391 for ln(NLR), 3.196 for ln(dNLR), and 2.537 for ln(DAR) (Table 4). Age remained independently associated with mortality in all models, while lower eGFR was associated with mortality in seven of the eight models. Diabetes mellitus was independently associated with mortality in the NLR model, and male sex was significant only in the LAR model. CAD/IHD was not independently associated with mortality in any of the adjusted models. Model-specific sample sizes, numbers of deaths, and goodness-of-fit measures are provided in Supplementary Table S6.

### 3.5. Missing Comorbidity Data and Sensitivity Analyses

Comorbidity information was complete for 1199 patients (61.4%) and incomplete for 753 (38.6%). Patients with complete and incomplete data were similar with respect to age, sex, ICU admission, CRP, neutrophil count, and LDH. However, mortality was higher among patients with complete comorbidity information (5.1% vs. 2.5%, *p* = 0.005), and significant differences were observed in albumin, lymphocyte count, eGFR, and D-dimer (Supplementary Table S7).

To assess the robustness of the primary findings, reduced multivariable models adjusted for age, sex, and eGFR were performed using the larger available samples and excluding diabetes mellitus and CAD/IHD from the adjustment set. All eight indices remained independently associated with mortality (all *p* < 0.001), with model-specific sample sizes ranging from 1847 to 1951 patients and 67–80 deaths (Supplementary Table S8). These findings were directionally consistent with the primary complete-case models.

### 3.6. Internal Validation

Bootstrap internal validation using 1000 resamples demonstrated minimal optimism in the discriminatory performance of the leading indices. Optimism-corrected AUCs were 0.886 for CAR, 0.886 for CLR, 0.862 for CPR, and approximately 0.857 for PNI. In contrast, the bootstrap distributions of the Youden-derived thresholds were wider, and optimism-corrected sensitivities were generally lower than the apparent estimates. For example, the bootstrap median threshold was 1.662 (2.5th–97.5th percentile, 0.901–2.692) for CAR and 41.020 (34.216–124.043) for CLR. These findings indicate relatively stable overall discrimination but greater uncertainty in the exact data-derived thresholds (Supplementary Table S10).

## 4. Discussion

In this retrospective cohort of 1952 hospitalized patients with COVID-19, several routinely available hematologic, inflammatory, biochemical, and nutritional indices were associated with in-hospital mortality. In the initial available-case ROC analyses, CAR and CLR showed the highest numerical discriminatory performance, each with an AUC of 0.870, followed by PNI (AUC 0.857) and CPR (AUC 0.852), while DAR, UAR, LAR, NLR, and dNLR also demonstrated meaningful prognostic discrimination. In multivariable analyses adjusted for age, sex, diabetes mellitus, CAD/IHD, and renal function, the selected indices remained independently associated with mortality. These findings suggest that composite laboratory-derived indices reflecting systemic inflammation, immune-cell alterations, nutritional status, tissue injury, renal dysfunction, and coagulation activity may provide complementary prognostic information in hospitalized patients with COVID-19.

Our findings are broadly consistent with previous studies evaluating inflammatory and composite biomarkers in COVID-19. NLR has been one of the most extensively studied indices, with meta-analyses showing that elevated admission NLR is associated with greater disease severity and mortality, with reported AUC values for mortality prediction of approximately 0.86–0.90 [1,10,11]. More recent studies have confirmed its prognostic relevance. Sayed et al. found that both NLR and SII were useful for identifying patients at increased risk of severe or fatal COVID-19 [12], while Harti et al. reported significant differences in NLR, SII, lymphocyte counts, and platelet-related inflammatory indices between survivors and non-survivors [13]. Additional evidence has similarly supported the prognostic value of NLR across different clinical settings [14]. In our cohort, NLR also showed good discrimination for in-hospital mortality (AUC 0.807), although its numerical performance was lower than that of CAR, CLR, PNI, and CPR.

These findings are consistent with previous reports showing that CAR is associated with COVID-19 mortality. In a retrospective cohort of 275 hospitalized patients, higher CAR was independently associated with in-hospital death [15], while a meta-analysis of 32 studies involving 12,445 patients confirmed significantly higher CAR values among non-survivors [16]. Similarly, the CLEAR COVID study reported markedly higher CLR values among deceased patients and an AUC of approximately 0.80 for mortality discrimination [17]. In our cohort, CLR demonstrated an AUC of 0.870, supporting its potential prognostic value.

PNI also showed strong discriminatory performance in our cohort (AUC 0.857), consistent with previous studies and meta-analyses linking lower PNI values with greater COVID-19 severity and mortality [5,6]. Reddy et al. similarly reported prognostic value for PNI and SII in assessing disease severity [18]. More recent studies have also highlighted the prognostic relevance of other nutritional-inflammatory indices, including HALP and CALLY, in patients with COVID-19 [19,20]. Although these indices were not evaluated in the present study, their findings support the broader concept that combined nutritional and inflammatory markers may provide useful prognostic information. This is consistent with the comparatively strong performance of PNI, CAR, and CLR in our cohort.

Because PNI integrates serum albumin and lymphocyte count, its prognostic value may reflect nutritional impairment, systemic inflammation, and immune dysregulation. Other albumin-based indices have also shown prognostic value in COVID-19. The blood urea nitrogen-to-albumin ratio has been associated with in-hospital mortality [21], while an elevated D-dimer-to-albumin ratio has been linked to severe disease and death [22]. Together, these findings support the potential value of composite indices that integrate inflammation with nutritional, renal, immune, or coagulation-related abnormalities.

Motloch et al. showed that cardiovascular and endothelial biomarkers, including VCAM-1, soluble ST2, and high-sensitivity troponin I, were associated with short- and long-term mortality in patients hospitalized with COVID-19 pneumonia [23]. These findings highlight the prognostic relevance of endothelial and cardiovascular injury. In contrast, the indices evaluated in our study are derived from routinely available laboratory measurements and may therefore offer a simpler and more accessible approach to early risk stratification.

The strong performance of CAR, CLR, CPR, and other albumin-based indices is biologically plausible in COVID-19. Severe SARS-CoV-2 infection is characterized by systemic inflammation, lymphocyte depletion, endothelial dysfunction, and increased vascular permeability. Lymphopenia has been associated with poor prognosis and may reflect impaired lymphocyte proliferation, enhanced apoptosis, and broader immune dysregulation [24]. Hypoalbuminemia is likewise associated with severe disease and mortality [25] and may result from the inflammation-related suppression of albumin synthesis, increased catabolism, and vascular leakage [26]. CAR therefore combines elevation of a positive acute-phase reactant with the reduction of a negative acute-phase protein, while CLR integrates systemic inflammation with lymphocyte depletion. These mechanisms may partly explain the strong discrimination observed for CAR and CLR in our cohort, with PNI showing similarly favorable performance.

An important limitation of CRP-based indices is that CRP is not specific to SARS-CoV-2-related inflammation and may also increase in the presence of bacterial coinfection or secondary bacterial infection [27]. Accordingly, elevated CAR, CLR, and CPR values may partly reflect concomitant bacterial infection in some patients. Because systematic data on admission procalcitonin levels, microbiological cultures, and antibiotic treatment were unavailable for the entire cohort, bacterial coinfection could not be included in the multivariable analyses. This potential residual confounding should therefore be considered when interpreting the prognostic performance of CRP-based indices.

An important finding was that the selected indices remained independently associated with in-hospital mortality after adjustment for age, sex, diabetes mellitus, CAD/IHD, and renal function. Age remained a consistent independent predictor across all models, in line with the well-established association between advanced age and adverse COVID-19 outcomes [28], while lower eGFR was also associated with mortality in most models. Diabetes mellitus was significant in the NLR model and showed borderline associations in some others, whereas CAD/IHD was not independently associated with mortality after adjustment. These findings suggest that the evaluated indices provided prognostic information beyond conventional clinical covariates. Although ln(LAR) showed the largest adjusted odds ratio, OR magnitudes should not be directly compared across differently scaled log-transformed biomarkers; the relative value of the indices is better interpreted together with discrimination, calibration, and overall model performance.

From a clinical perspective, the main advantage of these indices is their simplicity and accessibility. Because they are derived from routinely obtained hematologic and biochemical parameters, they require no additional testing or specialized equipment and may therefore be useful for rapid risk stratification, particularly in resource-limited settings. Recent studies have similarly highlighted the diagnostic and prognostic value of routine hematologic measurements and their derived indices in COVID-19 [29,30]. By comparing 18 laboratory-derived indices within the same cohort, our study showed that CAR and CLR had the highest numerical discrimination, while PNI also performed strongly and several other indices remained independently associated with mortality. These markers may therefore serve as complementary tools for identifying higher-risk patients, but they should not replace comprehensive clinical assessment, physiological monitoring, imaging, or validated prediction models.

Several limitations should be considered when interpreting these findings. First, the retrospective single-center design limits causal inference and may introduce selection and information bias. Second, comorbidity data were incomplete for a substantial proportion of the cohort, resulting in variable complete-case sample sizes. Although comparisons between patients with complete and incomplete comorbidity information and reduced-model sensitivity analyses supported the robustness of the principal associations, selection bias related to missing data cannot be excluded. Third, only 80 in-hospital deaths occurred, which limits the precision of some estimates and particularly the stability of data-derived thresholds. The Youden-based cut-off values were derived within the same cohort and, although bootstrap internal validation demonstrated relatively stable discrimination, the thresholds themselves showed greater variability and should therefore be regarded as exploratory rather than clinically established decision limits. Standardized viral-load data, detailed and consistently recorded antiviral and corticosteroid treatment information, and prealbumin measurements were not available for the entire cohort. Systematic information on bacterial coinfection, including admission procalcitonin levels, microbiological culture results, and antibiotic treatment, was also unavailable and may have influenced CRP-based indices such as CAR, CLR, and CPR. In addition, not all potentially relevant clinical severity measures were available. Finally, the cohort was restricted to patients hospitalized between 16 March and 4 July 2020 and therefore represents the early phase of the pandemic. The findings may not be directly generalizable to later SARS-CoV-2 variants, vaccinated populations, or contemporary treatment settings, and external validation in independent cohorts remains necessary.

## 5. Conclusions

In this large cohort of hospitalized patients with COVID-19, several routinely available hematologic, inflammatory, biochemical, and nutritional indices were associated with in-hospital mortality. CAR and CLR showed the highest numerical discrimination, while PNI also demonstrated strong performance. Several selected indices remained independently associated with mortality after adjustment for relevant clinical covariates. These findings support the potential value of laboratory-derived composite indices as adjunctive markers for risk stratification rather than stand-alone prognostic tools. Further prospective studies and external validation are required to determine their clinical utility and to confirm the reproducibility of the proposed cut-off values before routine implementation.

## Figures and Tables

**Table 1:** Baseline demographic, clinical, laboratory, and inflammatory index characteristics.

Variable	Overall Cohort
**Demographic and clinical characteristics**	
**Age, years**	48 (35–61)
**Male sex, n (%)**	978 (50.1)
**Female sex, n (%)**	974 (49.9)
**Hospital stay, days**	6 (4–8)
**ICU admission, n (%)**	176 (9.0)
**In-hospital mortality, n (%)**	80 (4.1)
**Comorbidities †**	
**Hypertension, n/N (%)**	302/1199 (25.2)
**Diabetes mellitus, n/N (%)**	118/1199 (9.8)
**CAD/IHD, n/N (%)**	136/1199 (11.3)
**Asthma, n/N (%)**	103/1199 (8.6)
**COPD, n/N (%)**	49/1199 (4.1)
**Other comorbidity, n/N (%)**	73/1199 (6.1)
**Laboratory parameters**	
**WBC, ×10^3^/µL**	6.11 (4.70–8.12)
**Neutrophils, ×10^3^/µL**	4.00 (2.90–5.80)
**Lymphocytes, ×10^3^/µL**	1.40 (1.02–1.89)
**Hemoglobin, g/dL**	13.9 (12.7–15.0)
**Hematocrit, %**	43.1 (39.7–46.0)
**Platelets, ×10^3^/µL**	209 (168–255)
**RDW-CV, %**	13.3 (12.8–14.0)
**MPV, fL**	10.1 (9.4–10.8)
**Glucose, mg/dL**	107.7 (96.0–129.0)
**Urea, mg/dL**	27.0 (21.0–36.0)
**Creatinine, mg/dL**	0.81 (0.71–0.97)
**Sodium, mmol/L**	138 (136–139)
**Potassium, mmol/L**	4.0 (4.0–4.0)
**Calcium, mg/dL**	9.0 (8.0–9.0)
**Albumin, g/L**	39 (36–42)
**AST, U/L**	24 (18–34)
**ALT, U/L**	21 (15–33)
**LDH, U/L**	242 (197–305)
**CRP, mg/L**	18.0 (3.8–53.8)
**Inflammatory/composite indices**	
**NLR**	2.82 (1.88–4.40)
**PLR**	143.7 (110.7–197.7)
**SII**	567.0 (356.6–982.7)
**NPR**	0.019 (0.014–0.027)
**RPR**	0.065 (0.053–0.081)
**MPR**	0.048 (0.038–0.062)
**dNLR**	2.07 (1.45–3.08)
**CAR**	0.45 (0.10–1.46)
**CLR**	13.10 (2.81–44.41)
**LAR**	6.21 (4.79–8.24)
**UAR**	0.70 (0.52–0.97)
**LLR**	176.3 (117.2–275.0)
**CPR**	0.089 (0.019–0.269)
**RLR**	9.64 (7.09–13.30)
**FAR**	4.79 (2.00–10.56)
**DAR**	4.42 (2.74–7.84)
**PNI**	46.5 (42.4–51.1)
**AST/ALT ratio**	1.15 (0.88–1.48)

Abbreviations: ICU, intensive care unit; CAD/IHD, coronary artery disease/ischemic heart disease; COPD, chronic obstructive pulmonary disease; WBC, white blood cell count; RDW-CV, red cell distribution width–coefficient of variation; MPV, mean platelet volume; AST, aspartate aminotransferase; ALT, alanine aminotransferase; LDH, lactate dehydrogenase; CRP, C-reactive protein; NLR, neutrophil-to-lymphocyte ratio; PLR, platelet-to-lymphocyte ratio; SII, systemic immune-inflammation index; NPR, neutrophil-to-platelet ratio; RPR, RDW-to-platelet ratio; MPR, MPV-to-platelet ratio; dNLR, derived neutrophil-to-lymphocyte ratio; CAR, CRP-to-albumin ratio; CLR, CRP-to-lymphocyte ratio; LAR, LDH-to-albumin ratio; UAR, urea-to-albumin ratio; LLR, LDH-to-lymphocyte ratio; CPR, CRP-to-platelet ratio; RLR, RDW-to-lymphocyte ratio; FAR, fibrinogen-to-albumin ratio; DAR, D-dimer-to-albumin ratio; PNI, prognostic nutritional index. Data are presented as median (interquartile range [IQR]) for continuous variables and n (%) or n/N (%) for categorical variables, as appropriate. † Comorbidity data were available for 1199 patients.

**Table 2:** Comparison of baseline characteristics between survivors and non-survivors.

Variable	Survivors (*n* = 1872)	Non-Survivors (*n* = 80)	*p* Value
**Demographic and comorbidity characteristics**			
**Male sex, n (%)**	935 (49.9)	43 (53.8)	0.505
**Hypertension, n/N (%)**	273/1138 (24.0)	29/61 (47.5)	<0.001
**Diabetes mellitus, n/N (%)**	103/1138 (9.1)	15/61 (24.6)	<0.001
**CAD/IHD, n/N (%)**	122/1138 (10.7)	14/61 (23.0)	0.003
**Asthma, n/N (%)**	101/1138 (8.9)	2/61 (3.3)	0.129
**COPD, n/N (%)**	48/1138 (4.2)	1/61 (1.6)	0.510 *
**Continuous characteristics**			
**Age, years**	47 (34–60)	72.5 (64–80)	<0.001
**Hospital stay, days**	5 (3–7)	8 (4–11.3)	<0.001
**WBC, ×10^3^/µL**	6.07 (4.65–7.94)	9.65 (6.16–12.97)	<0.001
**Neutrophils, ×10^3^/µL**	3.94 (2.86–5.67)	7.14 (4.69–10.75)	<0.001
**Lymphocytes, ×10^3^/µL**	1.43 (1.05–1.90)	0.80 (0.62–1.34)	<0.001
**Hemoglobin, g/dL**	14.0 (12.7–15.0)	13.0 (11.5–14.4)	<0.001
**Hematocrit, %**	43.2 (39.8–46.1)	40.5 (36.1–44.2)	<0.001
**Platelets, ×10^3^/µL**	209 (168–255)	218 (172.8–284.5)	0.370
**RDW-CV, %**	13.3 (12.8–14.0)	13.9 (13.3–15.3)	<0.001
**MPV, fL**	10.1 (9.4–10.8)	10.3 (9.4–11.2)	0.244
**Glucose, mg/dL**	107 (96–127)	137 (107.8–184.5)	<0.001
**Urea, mg/dL**	27 (21–35)	56.5 (34–89)	<0.001
**Creatinine, mg/dL**	0.81 (0.71–0.96)	1.13 (0.87–1.58)	<0.001
**Sodium, mmol/L**	138 (136–139)	136 (133–138)	<0.001
**Potassium, mmol/L**	4.0 (4.0–4.0)	4.0 (4.0–5.0)	0.052
**Calcium, mg/dL**	9.0 (8.0–9.0)	8.0 (8.0–8.8)	<0.001
**Albumin, g/L**	39 (36–43)	32.5 (28.8–37.0)	<0.001
**AST, U/L**	24 (18–34)	35 (24–56)	<0.001
**ALT, U/L**	21 (15–33)	22.5 (15–32.3)	0.780
**LDH, U/L**	239 (196–296)	397 (293.8–545.8)	<0.001
**CRP, mg/L**	16.5 (3.6–47.8)	130.5 (71.5–193.1)	<0.001

Abbreviations: CAD/IHD, coronary artery disease/ischemic heart disease; COPD, chronic obstructive pulmonary disease; WBC, white blood cell count; RDW-CV, red cell distribution width–coefficient of variation; MPV, mean platelet volume; AST, aspartate aminotransferase; ALT, alanine aminotransferase; LDH, lactate dehydrogenase; CRP, C-reactive protein. Data are presented as median (interquartile range [IQR]) for continuous variables and n (%) or n/N (%) for categorical variables, as appropriate. Continuous variables were compared using the Mann–Whitney U test. Categorical variables were compared using Pearson’s chi-square test or Fisher’s exact test when expected cell counts were insufficient. Comorbidity data were available for 1199 patients, including 1138 survivors and 61 non-survivors. * Fisher’s exact test.

**Table 3:** ROC analysis of inflammatory indices for in-hospital mortality.

Index	AUC	Optimal Cut-Off	Sensitivity (%)	Specificity (%)	Youden Index
**CAR**	0.870	1.644	82.4	80.2	0.626
**CLR**	0.870	41.031	86.3	75.4	0.617
**PNI**	0.857	≤43.975	89.6	68.0	0.576
**CPR**	0.852	0.2409	86.3	73.5	0.598
**DAR**	0.829	10.196	72.5	84.6	0.571
**UAR**	0.823	1.058	68.6	82.5	0.511
**LAR**	0.815	7.803	80.4	72.9	0.533
**NLR**	0.807	3.972	80.4	73.1	0.535
**dNLR**	0.797	2.690	82.4	70.0	0.524
**LLR**	0.795	319.974	64.7	82.9	0.476
**FAR**	0.782	8.462	78.4	71.2	0.496
**SII**	0.759	910.659	76.5	75.0	0.515
**RLR**	0.759	12.995	74.5	73.2	0.477
**AST/ALT ratio**	0.734	1.423	67.5	73.2	0.407
**NPR**	0.727	0.0232	72.5	66.0	0.385
**PLR**	0.712	187.451	70.6	72.9	0.435
**RPR**	0.519	0.1467	9.8	98.6	0.084
**MPR**	0.501	0.0928	13.7	94.9	0.086

Abbreviations: AUC, area under the receiver operating characteristic curve; CAR, C-reactive protein-to-albumin ratio; CLR, C-reactive protein-to-lymphocyte ratio; PNI, prognostic nutritional index; CPR, C-reactive protein-to-platelet ratio; DAR, D-dimer-to-albumin ratio; UAR, urea-to-albumin ratio; LAR, lactate dehydrogenase-to-albumin ratio; NLR, neutrophil-to-lymphocyte ratio; dNLR, derived neutrophil-to-lymphocyte ratio; LLR, lactate dehydrogenase-to-lymphocyte ratio; FAR, fibrinogen-to-albumin ratio; SII, systemic immune-inflammation index; RLR, red cell distribution width-to-lymphocyte ratio; AST, aspartate aminotransferase; ALT, alanine aminotransferase; NPR, neutrophil-to-platelet ratio; PLR, platelet-to-lymphocyte ratio; RPR, red cell distribution width-to-platelet ratio; MPR, mean platelet volume-to-platelet ratio. Receiver operating characteristic (ROC) analysis was used to evaluate the discriminatory ability of each index for in-hospital mortality. Optimal cut-off values were determined using the maximum Youden index, calculated as sensitivity + specificity − 1.

**Table 4:** Multivariable logistic regression analyses of selected laboratory-derived indices for in-hospital mortality, additionally adjusted for renal function.

Model	N	Deaths, n	Predictor	Adjusted OR	95% CI	*p* Value
**CLR model**	1188	58	Age, per year	1.063	1.034–1.092	<0.001
			Male sex	1.195	0.603–2.369	0.610
			Diabetes mellitus	2.001	0.896–4.466	0.090
			CAD/IHD	1.307	0.585–2.923	0.514
			eGFR, per 1 mL/min/1.73 m^2^	0.976	0.964–0.989	<0.001
			ln(CLR)	3.590	2.517–5.120	<0.001
**CAR model**	1193	61	Age, per year	1.062	1.035–1.090	<0.001
			Male sex	1.501	0.781–2.883	0.223
			Diabetes mellitus	1.428	0.653–3.123	0.372
			CAD/IHD	1.291	0.588–2.834	0.524
			eGFR, per 1 mL/min/1.73 m^2^	0.975	0.963–0.987	<0.001
			ln(CAR)	4.399	2.876–6.729	<0.001
**CPR model**	1189	60	Age, per year	1.055	1.028–1.081	<0.001
			Male sex	1.217	0.633–2.339	0.555
			Diabetes mellitus	1.289	0.595–2.793	0.520
			CAD/IHD	1.183	0.546–2.563	0.671
			eGFR, per 1 mL/min/1.73 m^2^	0.971	0.959–0.983	<0.001
			ln(CPR)	3.552	2.401–5.255	<0.001
**LAR model**	1198	61	Age, per year	1.074	1.046–1.102	<0.001
			Male sex	2.154	1.113–4.168	0.023
			Diabetes mellitus	1.530	0.702–3.332	0.285
			CAD/IHD	1.054	0.489–2.274	0.893
			eGFR, per 1 mL/min/1.73 m^2^	0.972	0.960–0.984	<0.001
			ln(LAR)	17.216	8.207–36.115	<0.001
**UAR model**	1198	61	Age, per year	1.067	1.042–1.093	<0.001
			Male sex	1.361	0.735–2.523	0.327
			Diabetes mellitus	1.564	0.762–3.209	0.223
			CAD/IHD	0.852	0.414–1.755	0.664
			eGFR, per 1 mL/min/1.73 m^2^	0.995	0.979–1.012	0.573
			ln(UAR)	4.195	2.123–8.288	<0.001
**NLR model**	1165	50	Age, per year	1.069	1.041–1.098	<0.001
			Male sex	1.563	0.789–3.098	0.200
			Diabetes mellitus	2.444	1.148–5.201	0.020
			CAD/IHD	1.027	0.477–2.215	0.945
			eGFR, per 1 mL/min/1.73 m^2^	0.980	0.967–0.993	0.002
			ln(NLR)	3.391	2.109–5.452	<0.001
**dNLR model**	1168	52	Age, per year	1.066	1.039–1.093	<0.001
			Male sex	1.925	0.998–3.713	0.051
			Diabetes mellitus	2.050	0.988–4.254	0.054
			CAD/IHD	0.918	0.434–1.943	0.822
			eGFR, per 1 mL/min/1.73 m^2^	0.975	0.963–0.988	<0.001
			ln(dNLR)	3.196	1.873–5.454	<0.001
**DAR model**	1115	55	Age, per year	1.062	1.036–1.089	<0.001
			Male sex	1.709	0.891–3.278	0.107
			Diabetes mellitus	2.096	0.986–4.456	0.054
			CAD/IHD	0.790	0.366–1.703	0.547
			eGFR, per 1 mL/min/1.73 m^2^	0.978	0.967–0.990	<0.001
			ln(DAR)	2.537	1.846–3.487	<0.001

Abbreviations: OR, odds ratio; CI, confidence interval; CAD/IHD, coronary artery disease/ischemic heart disease; eGFR, estimated glomerular filtration rate; ln, natural logarithm; CLR, C-reactive protein-to-lymphocyte ratio; CAR, C-reactive protein-to-albumin ratio; CPR, C-reactive protein-to-platelet ratio; LAR, lactate dehydrogenase-to-albumin ratio; UAR, urea-to-albumin ratio; NLR, neutrophil-to-lymphocyte ratio; dNLR, derived neutrophil-to-lymphocyte ratio; DAR, D-dimer-to-albumin ratio. eGFR was calculated using the 2021 CKD-EPI creatinine equation. Multivariable models were performed using complete cases for the variables included in each model; therefore, analytical sample sizes and numbers of mortality events varied across models.

## Data Availability

The data supporting the findings of this study are available from the corresponding author upon reasonable request.

## Supplementary Materials

The following supporting information can be downloaded at: https://www.mdpi.com/article/10.3390/jcm15218419/s1, Table S1: Formulas used for calculation of hematologic and inflammatory indices; Table S2: Univariate logistic regression of clinical predictors of in-hospital mortality; Table S3: Univariate logistic regression of natural-log-transformed indices; Table S4: Univariate logistic regression of indices on the original scale; Table S5: Model fit and calibration of univariate log-transformed index models; Table S6: Sample size, model fit, and calibration of multivariable logistic regression models; Table S7: Comparison of patients with complete and incomplete comorbidity data; Table S8: Sensitivity analyses using reduced multivariable models adjusted for age, sex, and eGFR; Table S9: Pairwise DeLong comparisons of the leading laboratory-derived indices in the common complete-case cohort; Table S10: Bootstrap internal validation of ROC discrimination and Youden-derived thresholds for the leading laboratory-derived indices.

## Abbreviations

The following abbreviations are used in this manuscript: Abbreviations: ALT, alanine aminotransferase; aOR, adjusted odds ratio; AST, aspartate aminotransferase; AUC, area under the receiver operating characteristic curve; CAD, coronary artery disease; CAR, C-reactive protein-to-albumin ratio; CI, confidence interval; CLR, C-reactive protein-to-lymphocyte ratio; COPD, chronic obstructive pulmonary disease; COVID-19, coronavirus disease 2019; CPR, C-reactive protein-to-platelet ratio; CRP, C-reactive protein; DAR, D-dimer-to-albumin ratio; dNLR, derived neutrophil-to-lymphocyte ratio; FAR, fibrinogen-to-albumin ratio; ICU, intensive care unit; IHD, ischemic heart disease; LAR, lactate dehydrogenase-to-albumin ratio; LDH, lactate dehydrogenase; LLR, lactate dehydrogenase-to-lymphocyte ratio; ln, natural logarithm; MPR, mean platelet volume-to-platelet ratio; MPV, mean platelet volume; NLR, neutrophil-to-lymphocyte ratio; NPR, neutrophil-to-platelet ratio; OR, odds ratio; PLR, platelet-to-lymphocyte ratio; PNI, prognostic nutritional index; RDW-CV, red cell distribution width–coefficient of variation; RLR, red cell distribution width-to-lymphocyte ratio; ROC, receiver operating characteristic; RPR, red cell distribution width-to-platelet ratio; RT-PCR, reverse transcription polymerase chain reaction; SARS-CoV-2, severe acute respiratory syndrome coronavirus 2; SII, systemic immune-inflammation index; UAR, urea-to-albumin ratio; WBC, white blood cell count.
