# Supplementary material for: Comparative Prognostic Performance of 18 Routine Laboratory-Derived Indices for In-Hospital Mortality in Patients with COVID-19
Source: J Clin Med. 2026 Oct 29;15(21):8419. doi: 10.3390/jcm15218419 (PMC13763979; doi:10.3390/jcm15218419)
Supplement: Supplementary file 1 [file jcm-15-08419-s001.zip › jcm-4643718-supplementary.pdf]

Supplementary Table S1. Formulas used for calculation of hematologic and inflammatory indices.

Index	Formula	Index	Formula
**NLR**	Neutrophils / Lymphocytes	CAR	CRP / Albumin
**PLR**	Platelets / Lymphocytes	CLR	CRP / Lymphocytes
**SII**	(Platelets × Neutrophils) / Lymphocytes	LAR	LDH / Albumin
**NPR**	Neutrophils / Platelets	UAR	Urea / Albumin
**RPR**	RDW / Platelets	LLR	LDH / Lymphocytes
**MPR**	MPV / Platelets	CPR	CRP / Platelets
**dNLR**	Neutrophils / (WBC − Neutrophils)	RLR	RDW / Lymphocytes
**FAR**	Fibrinogen / Albumin	DAR	D-dimer / Albumin
**PNI**	10 × Albumin (g/dL) + 0.005 × Lymphocyte count (/mm³)	AST/ALT ratio	AST / ALT

Abbreviations: NLR, neutrophil-to-lymphocyte ratio; PLR, platelet-to-lymphocyte ratio; SII, systemic immune-inflammation index; NPR, neutrophil-to-platelet ratio; RPR, red cell distribution width-to-platelet ratio; MPR, mean platelet volume-to-platelet ratio; dNLR, derived neutrophil-to-lymphocyte ratio; CAR, C-reactive protein-to-albumin ratio; CLR, C-reactive protein-to-lymphocyte ratio; LAR, lactate dehydrogenase-to-albumin ratio; UAR, urea-to-albumin ratio; LLR, lactate dehydrogenase-to-lymphocyte ratio; CPR, C-reactive protein-to-platelet ratio; RLR, red cell distribution width-to-lymphocyte ratio; FAR, fibrinogen-to-albumin ratio; DAR, D-dimer-to-albumin ratio; PNI, prognostic nutritional index; WBC, white blood cell count; CRP, C-reactive protein; LDH, lactate dehydrogenase; RDW, red cell distribution width; MPV, mean platelet volume; AST, aspartate aminotransferase; ALT, alanine aminotransferase.

Supplementary Table S2. Univariate logistic regression of clinical predictors of in-hospital mortality

Predictor	B	Crude OR	95% CI	p value
**Male sex**	0.152	1.165	0.744–1.824	0.506
**Age, per year**	0.089	1.094	1.075–1.113	<0.001
**Hypertension**	1.055	2.871	1.706–4.833	<0.001
**Diabetes mellitus**	1.187	3.277	1.768–6.073	<0.001
**CAD/IHD**	0.909	2.481	1.327–4.637	0.004
**Asthma**	−1.055	0.348	0.084–1.446	0.146
**COPD**	−0.972	0.378	0.051–2.789	0.340
**Number of comorbidities**	0.606	1.834	1.462–2.301	<0.001

Abbreviations: OR, odds ratio; CI, confidence interval; CAD/IHD, coronary artery disease/ischemic heart disease; COPD, chronic obstructive pulmonary disease.

Supplementary Table S3. Univariate logistic regression of natural-log-transformed indices

Index	B	OR	95% CI	p value
**ln(NLR)**	1.665	5.287	3.764–7.425	<0.001
**ln(PLR)**	1.780	5.932	3.921–8.976	<0.001
**ln(SII)**	1.290	3.632	2.704–4.877	<0.001
**ln(NPR)**	1.678	5.356	3.305–8.680	<0.001
**ln(RPR)**	0.382	1.466	0.761–2.825	0.253
**ln(MPR)**	0.100	1.106	0.618–1.977	0.735
**ln(dNLR)**	1.703	5.491	3.781–7.973	<0.001
**ln(CAR)**	1.441	4.224	3.184–5.604	<0.001
**ln(CLR)**	1.280	3.597	2.831–4.570	<0.001
**ln(LAR)**	2.726	15.276	9.498–24.569	<0.001
**ln(UAR)**	2.030	7.615	5.403–10.735	<0.001
**ln(LLR)**	1.978	7.229	5.056–10.334	<0.001
**ln(CPR)**	1.290	3.631	2.780–4.744	<0.001
**ln(RLR)**	2.138	8.482	5.256–13.688	<0.001
**ln(FAR)**	1.006	2.736	2.137–3.503	<0.001
**ln(DAR)**	1.145	3.141	2.549–3.870	<0.001
**ln(PNI)**	−7.956	<0.001	<0.001–0.001	<0.001
**ln(AST/ALT)**	2.290	9.878	5.512–17.702	<0.001

Abbreviations: ln, natural logarithm; OR, odds ratio; CI, confidence interval; NLR, neutrophil-to-lymphocyte ratio; PLR, platelet-to-lymphocyte ratio; SII, systemic immune-inflammation index; NPR, neutrophil-to-platelet ratio; RPR, red cell distribution width-to-platelet ratio; MPR, mean platelet volume-to-platelet ratio; dNLR, derived neutrophil-to-lymphocyte ratio; CAR, C-reactive protein-to-albumin ratio; CLR, C-reactive protein-to-lymphocyte ratio; LAR, lactate dehydrogenase-to-albumin ratio; UAR, urea-to-albumin ratio; LLR, lactate dehydrogenase-to-lymphocyte ratio; CPR, C-reactive protein-to-platelet ratio; RLR, red cell distribution width-to-lymphocyte ratio; FAR, fibrinogen-to-albumin ratio; DAR, D-dimer-to-albumin ratio; PNI, prognostic nutritional index; AST, aspartate aminotransferase; ALT, alanine aminotransferase.

Supplementary Table S4. Univariate logistic regression of indices on the original scale

Index	B	OR	95% CI	p value
**NLR**	0.154	1.167	1.122–1.213	<0.001
**PLR**	0.004	1.004	1.003–1.006	<0.001
**SII**	<0.001	1.000	1.000–1.001	<0.001
**NPR**	43.664	9.18×10^{18}	2.62×10^{13}–3.22×10^{24}	<0.001
**RPR**	5.042	154.727	1.075–22,264.243	0.047
**MPR**	4.313	74.682	0.152–36,629.752	0.172
**dNLR**	0.245	1.278	1.193–1.370	<0.001
**CAR**	0.477	1.612	1.485–1.749	<0.001
**CLR**	0.008	1.008	1.006–1.010	<0.001
**LAR**	0.173	1.189	1.147–1.233	<0.001
**UAR**	0.927	2.527	2.102–3.038	<0.001
**LLR**	0.004	1.004	1.003–1.004	<0.001
**CPR**	2.354	10.531	6.571–16.877	<0.001
**RLR**	0.071	1.074	1.048–1.101	<0.001
**FAR**	0.039	1.040	1.028–1.052	<0.001
**DAR**	0.016	1.016	1.010–1.022	<0.001
**PNI**	−0.213	0.808	0.778–0.840	<0.001
**AST/ALT ratio**	1.314	3.721	2.692–5.142	<0.001

Abbreviations: OR, odds ratio; CI, confidence interval; NLR, neutrophil-to-lymphocyte ratio; PLR, platelet-to-lymphocyte ratio; SII, systemic immune-inflammation index; NPR, neutrophil-to-platelet ratio; RPR, red cell distribution width-to-platelet ratio; MPR, mean platelet volume-to-platelet ratio; dNLR, derived neutrophil-to-lymphocyte ratio; CAR, C-reactive protein-to-albumin ratio; CLR, C-reactive protein-to-lymphocyte ratio;

LAR, lactate dehydrogenase-to-albumin ratio; UAR, urea-to-albumin ratio; LLR, lactate dehydrogenase-to-lymphocyte ratio; CPR, C-reactive protein-to-platelet ratio; RLR, red cell distribution width-to-lymphocyte ratio; FAR, fibrinogen-to-albumin ratio; DAR, D-dimer-to-albumin ratio; PNI, prognostic nutritional index; AST, aspartate aminotransferase; ALT, alanine aminotransferase.

Supplementary Table S5. Model fit and calibration of univariate log-transformed index models

Index	−2 Log likelihood	Nagelkerke R²	Hosmer–Lemeshow p
**ln(NLR)**	479.739	0.195	0.198
**ln(PLR)**	569.800	0.128	0.003
**ln(SII)**	495.343	0.153	<0.001
**ln(NPR)**	546.001	0.091	0.638
**ln(RPR)**	636.593	0.002	0.271
**ln(MPR)**	653.885	0.000	0.065
**ln(dNLR)**	509.934	0.160	0.301
**ln(CAR)**	474.512	0.317	0.046
**ln(CLR)**	450.110	0.333	0.642
**ln(LAR)**	515.420	0.259	0.378
**ln(UAR)**	517.740	0.255	0.939
**ln(LLR)**	507.752	0.245	0.168
**ln(CPR)**	501.394	0.264	0.821
**ln(RLR)**	537.641	0.160	0.113
**ln(FAR)**	464.824	0.164	0.012
**ln(DAR)**	489.720	0.241	0.170
**ln(PNI)**	497.622	0.262	0.641
**ln(AST/ALT)**	604.735	0.110	0.091

Abbreviations: ln, natural logarithm; NLR, neutrophil-to-lymphocyte ratio; PLR, platelet-to-lymphocyte ratio; SII, systemic immune-inflammation index; NPR, neutrophil-to-platelet ratio; RPR, red cell distribution width-to-platelet ratio; MPR, mean platelet volume-to-platelet ratio; dNLR, derived neutrophil-to-lymphocyte ratio; CAR, C-reactive protein-to-albumin ratio; CLR, C-reactive protein-to-lymphocyte ratio; LAR, lactate dehydrogenase-to-albumin ratio; UAR, urea-to-albumin ratio; LLR, lactate dehydrogenase-to-lymphocyte ratio; CPR, C-reactive protein-to-platelet ratio; RLR, red cell distribution width-to-lymphocyte ratio; FAR, fibrinogen-to-albumin ratio; DAR, D-dimer-to-albumin ratio; PNI, prognostic nutritional index; AST, aspartate aminotransferase; ALT, alanine aminotransferase.

Supplementary Table S6. Sample size, model fit, and calibration of multivariable logistic regression models

Model	n	Survivors	Non-survivors	−2 Log likelihood	Nagelkerke R²	Hosmer–Lemeshow p
**CLR**	1,188	1,130	58	248.960	0.511	0.997
**CAR**	1,193	1,132	61	268.449	0.493	<0.001
**CPR**	1,189	1,129	60	287.331	0.444	0.018
**LAR**	1,198	1,137	61	279.952	0.469	0.715
**UAR**	1,198	1,137	61	318.550	0.385	0.087
**NLR**	1,165	1,115	50	266.431	0.395	0.727
**dNLR**	1,168	1,116	52	292.197	0.353	0.200
**DAR**	1,115	1,060	55	279.485	0.408	0.952

Abbreviations: CLR, C-reactive protein-to-lymphocyte ratio; CAR, C-reactive protein-to-albumin ratio; CPR, C-reactive protein-to-platelet ratio; LAR, lactate dehydrogenase-to-albumin ratio; UAR, urea-to-albumin ratio; NLR, neutrophil-to-lymphocyte ratio; dNLR, derived neutrophil-to-lymphocyte ratio; DAR, D-dimer-to-albumin ratio; R^2, coefficient of determination.

Supplementary Table S7. Comparison of patients with complete and incomplete comorbidity information

Variable	Incomplete comorbidity data (n=753)	Complete comorbidity data (n=1,199)	p value
**Age, years**	48 (35–62)	48 (35–61)	0.738
**Male sex, n (%)**	391 (51.9)	587 (49.0)	0.202
**ICU admission, n (%)**	70 (9.3)	106 (8.8)	0.732
**In-hospital mortality, n (%)**	19 (2.5)	61 (5.1)	**0.005**
**CRP, mg/L**	19.3 (4.55–58.15)	16.9 (3.50–51.90)	0.099
**Albumin, g/L**	38 (35–41)	40 (37–43)	**<0.001**
**Lymphocyte count, ×10⁹/L**	1.35 (0.99–1.80)	1.44 (1.04–1.93)	**0.005**
**Neutrophil count, ×10⁹/L**	3.93 (2.85–5.73)	4.07 (2.89–5.87)	0.192
**LDH, U/L**	247 (198–309)	240 (196–301)	0.219
**eGFR, mL/min/1.73 m²**	99.4 (81.25–112.40)	101.9 (86.30–113.90)	**0.009**
**D-dimer**	162.5 (106.75–276.25)	176.0 (115.00–313.00)	**0.017**

Continuous variables are presented as median (25th–75th percentile) and were compared using the Mann–Whitney U test. Categorical variables are presented as n (%) and were compared using Pearson's chi-square test. Laboratory-variable denominators may differ from the overall group totals because of variable-specific missing data; notably, D-dimer was available for 734 patients in the incomplete-comorbidity group and 1,118 patients in the complete-comorbidity group. **Abbreviations:** CRP, C-reactive protein; ICU, intensive care unit; LDH, lactate dehydrogenase; eGFR, estimated glomerular filtration rate; IQR, interquartile range; Q1, 25th percentile; Q3, 75th percentile.

Supplementary Table S8. Reduced-model sensitivity analyses of selected laboratory-derived indices for in-hospital mortality

Index	N	Deaths, n	Adjusted OR	95% CI	p value	Nagelkerke R^2	Hosmer–Lemeshow p
**CLR**	1,936	76	**3.035**	2.317–3.975	**<0.001**	0.457	0.868
**CAR**	1,945	79	**3.278**	2.410–4.459	**<0.001**	0.435	0.163
**CPR**	1,939	78	**2.719**	2.022–3.656	**<0.001**	0.398	0.804
**LAR**	1,951	80	**11.211**	6.516–19.289	**<0.001**	0.433	0.982

**UAR**	1,951	80	**4.018**	2.234 – 7.226	**<0.001**	0.330	0.357
**NLR**	1,895	67	**3.084**	2.121 – 4.484	**<0.001**	0.360	0.918
**dNLR**	1,900	69	**3.214**	2.108 – 4.901	**<0.001**	0.343	0.966
**DAR**	1,847	74	**2.163**	1.699 – 2.755	**<0.001**	0.365	0.820

Each index was entered into a separate multivariable binary logistic regression model adjusted for age, sex, and eGFR. Diabetes mellitus and coronary artery disease/ischemic heart disease were omitted from these reduced models to minimize loss of observations related to incomplete comorbidity information. Right-skewed indices were natural-log transformed before regression. Model-specific N and mortality-event counts vary according to index availability. **Abbreviations:** CAR, C-reactive protein-to-albumin ratio; CLR, C-reactive protein-to-lymphocyte ratio; CPR, C-reactive protein-to-platelet ratio; DAR, D-dimer-to-albumin ratio; dNLR, derived neutrophil-to-lymphocyte ratio; eGFR, estimated glomerular filtration rate; LAR, lactate dehydrogenase-to-albumin ratio; NLR, neutrophil-to-lymphocyte ratio; OR, odds ratio; CI, confidence interval; UAR, urea-to-albumin ratio.

Supplementary Table S9. Pairwise comparison of ROC curves for CAR, CLR, CPR, and PNI in the common complete-case cohort

Comparison	AUC 1	AUC 2	ΔAUC	95% CI for ΔAUC	z statistic	p value
**CLR vs CAR**	0.888	0.886	0.001	−0.012 to 0.015	0.171	0.864
**CLR vs CPR**	0.888	0.862	0.025	0.005 to 0.045	2.456	0.014
**CLR vs PNI**	0.888	0.856	0.032	−0.005 to 0.068	1.705	0.088
**CAR vs CPR**	0.886	0.862	0.024	0.007 to 0.041	2.795	0.005
**CAR vs PNI**	0.886	0.856	0.030	−0.008 to 0.068	1.565	0.118
**CPR vs PNI**	0.862	0.856	0.006	−0.038 to 0.050	0.283	0.777

Footnote: Pairwise comparisons were performed using DeLong's test for correlated ROC curves. Analyses were restricted to the common complete-case cohort with available data for CAR, CLR, CPR, PNI, and in-hospital mortality (n=1,934; 76 deaths and 1,858 survivors). PNI was evaluated in the inverse direction because lower PNI values indicate greater mortality risk. A positive ΔAUC indicates a higher AUC for the first index listed in each comparison.

Abbreviations: AUC, area under the receiver operating characteristic curve; CAR, C-reactive protein-to-albumin ratio; CI, confidence interval; CLR, C-reactive protein-to-lymphocyte ratio; CPR, C-reactive protein-to-platelet ratio; PNI, prognostic nutritional index; ROC, receiver operating characteristic.

Supplementary Table S10. Bootstrap internal validation of ROC discrimination and Youden-derived thresholds for the leading laboratory-derived indices

Index	Apparent AUC	Mean AUC optimism	Optimism-corrected AUC	Apparent Youden cut-off	Bootstrap median cut-off (2.5th–97.5th percentile)	Apparent sensitivity (%)	Optimism-corrected sensitivity (%)	Apparent specificity (%)	Optimism-corrected specificity (%)
**CAR**	0.886	0.0004	0.886	1.649	1.662 (0.901–2.692)	82.9	76.6	80.3	80.1
**CLR**	0.888	0.0016	0.886	41.020	41.020 (34.216–124.043)	89.5	84.2	76.0	76.0
**CPR**	0.862	0.0008	0.862	0.275	0.288 (0.147–0.412)	81.6	75.5	78.1	77.9
**PNI***	0.856	−0.0007	0.857	≤43.975	≤43.550 (39.875–43.975)	89.5	83.8	68.0	68.0

Footnote: Internal validation was performed using 1,000 bootstrap resamples with replacement in the common complete-case cohort (n=1,934; 76 deaths and 1,858 survivors). In each bootstrap replicate, the optimal threshold was selected by maximizing the Youden index in the bootstrap sample and subsequently evaluated in observations not selected into that bootstrap sample (out-of-bag observations). Optimism was estimated as the mean difference between performance in the bootstrap sample and the corresponding out-of-bag performance. Optimism-corrected performance was obtained by subtracting the estimated optimism from the apparent performance in the original complete-case cohort. The 2.5th and 97.5th percentiles of the bootstrap-selected thresholds are shown as an empirical measure of threshold stability. *For PNI, lower values indicate greater mortality risk.

Abbreviations: AUC, area under the receiver operating characteristic curve; CAR, C-reactive protein-to-albumin ratio; CLR, C-reactive protein-to-lymphocyte ratio; CPR, C-reactive protein-to-platelet ratio; PNI, prognostic nutritional index; ROC, receiver operating characteristic.
